# A Narrative Review Examining the Utility of Interpersonal Synchrony for the Caregiver-Care Recipient Relationship in Alzheimer’s Disease and Related Dementias

**DOI:** 10.3389/fpsyg.2021.595816

**Published:** 2021-05-07

**Authors:** Angela Gifford, Vivien Marmelat, Janelle N. Beadle

**Affiliations:** ^1^Neuroscience and Behavior Graduate Program, Department of Psychology, University of Nebraska at Omaha, Omaha, NE, United States; ^2^Department of Biomechanics, University of Nebraska at Omaha, Omaha, NE, United States; ^3^Department of Gerontology, University of Nebraska at Omaha, Omaha, NE, United States

**Keywords:** caregiving, Alzheimer’s disease, prosocial behavior, well-being, empathy

## Abstract

The stressful nature of caring for an older adult with a chronic disease, such as Alzheimer’s disease (AD), can create barriers between the caregiver-care recipient, as they try to navigate their continuously changing social relationship. Interpersonal synchrony (i.e., matching or similarity of movement, emotions, hormones, or brain activity), is an innovative approach that could help to sustain caregiving relationship dynamics by promoting feelings of connection and empathy through shared behavior and experiences. This review investigates the current literature on interpersonal synchrony from an interdisciplinary perspective by examining interpersonal synchrony through psychological, neural, and hormonal measures across the adult lifespan. We then present a case for examining the degree to which interpersonal synchrony can be used to facilitate affiliation and well-being in the caregiver-care recipient relationship. We find that there is significant evidence in healthy adult populations that interpersonal synchrony can support affiliative feelings, prosocial behavior, and well-being. Characterizing the psychological, neural, and hormonal mechanisms of interpersonal synchrony is a first step towards laying the groundwork for the development of tools to support relational closeness and empathy in the caregiving context. Finally, we explore the strengths and limitations of using interpersonal synchrony to support relational well-being, and discuss possible avenues for future research.

## The Importance of Interpersonal Synchrony

Feelings of social connectedness are essential to the maintenance of health and well-being (Wilkins et al., 2010; Tunçgenç and Cohen, 2016). One mechanism to improve perceived social connection and relational well-being is engaging in interpersonal synchrony (Cirelli et al., 2014; Rennung and Göritz, 2016; Tunçgenç and Cohen, 2016). Interpersonal synchrony is defined as the matching of movements or sensations which elicit similarity in frequency or level of emotional experiences, hormones, and/or brain activity between two or more people (Rennung and Göritz, 2016). It should be noted that interpersonal synchrony differs from mimicry in that it does not require conscious awareness to complete a motion (Rennung and Göritz, 2016). Some examples of interpersonal synchrony include matching of movements between two individuals, for example, tapping one’s finger at the same rate, matching breathing between two people, subtle mirrored shifts in posture as seen in therapy-client relations, or shared feelings between individuals when viewing the same stimulus (Hove and Keller, 2015; Rennung and Göritz, 2016; Oesch, 2019; Wan and Fu, 2019). Group settings can also elicit forms of interpersonal synchrony, such as singing in groups or playing on a sports team (Tunçgenç and Cohen, 2016; Ikeda et al., 2017; Oesch, 2019).

Throughout the lifespan, interpersonal synchrony fosters social interaction and prosocial behaviors (Tunçgenç and Cohen, 2016; Oesch, 2019). For instance, in the relationship between mothers and children, interpersonal synchrony may help infants self-regulate their emotions (Busuito et al., 2019). In adults, interpersonal synchrony may increase social cohesion by shaping attitudes and behaviors towards others (Hove and Risen, 2009; Tunçgenç and Cohen, 2016). On the other hand, when we fall out of synchrony with others, our social bonds can be affected. For instance, individuals responded more positively to in-group members who completed inter-personal tasks with them than out-group members who did not perform these tasks with them (Hove and Risen, 2009; Tunçgenç and Cohen, 2016). Within romantic relationships, relationship stability and success over time are positively predicted by couples’ scores on interpersonal synchrony (Oesch, 2019).

Interpersonal synchrony can also elicit affiliative and prosocial actions, with interpersonal synchrony positively related to helping behaviors toward others (Valdesolo and DeSteno, 2011; Praszkier, 2016; Rennung and Göritz, 2016). It is theorized that synchronous actions may change perception, as synchronously linked individuals are viewed as being more similar to oneself, leading to more compassionate and altruistic behaviors (Valdesolo and DeSteno, 2011). These behaviors are closely tied to mechanisms of empathy, which is also a key component in nonverbal communication with others (Praszkier, 2016).

Although several studies have examined the role of interpersonal synchrony in the context of relationships in healthy adults (Liu et al., 2013; Roberts et al., 2013; Birditt et al., 2017), little research has examined the impact of interpersonal synchrony within caregiver-care recipient relationships in older adulthood, in particular, in Alzheimer’s disease (AD) and related dementias. This is important because Alzheimer’s disease is a growing public health issue as by 2050, it is predicted that 13.8 million Americans 65 years of age and older will have AD (Alzheimer’s Association, 2020). Individuals with Alzheimer’s disease require a significant amount of care and support throughout the disease process. In fact, it is estimated that there are at least 16 million Americans serving in the role of an unpaid caregiver to individuals with Alzheimer’s disease or related dementias (Alzheimer’s Association, 2020), with the majority being family members, such as a spouse, sibling, or child. The Family Caregiver Alliance defines informal, or unpaid, family caregivers as, “any relative, partner, friend, or neighbor who has a significant personal relationship with, and provides a broad range of assistance for, an older person or an adult with a chronic or disabling condition,” (Family Caregiver Alliance, 2020). On average, family caregivers typically provide approximately “21.9 h of care per week,” for individuals with Alzheimer’s disease or other forms of dementia,” (Alzheimer’s Association, 2015), and older caregivers typically provide care for “a spouse or partner,” (National Alliance for Caregiving and AARP, 2015.)

From a public health perspective, it is of particular importance to understand how patients with Alzheimer’s disease and other forms of dementia and their family caregivers are affected by interpersonal synchrony. For many, the caregiver-care recipient dynamic evolves over time, as neurodegeneration in the care recipient can result in personality changes, and socioemotional disturbances (Barsuglia et al., 2014; Marshall et al., 2018). Greater socioemotional and behavioral disturbances in Alzheimer’s disease and other forms of dementia have been linked to several mental health outcomes in caregivers, including depression, caregiver burnout, burden, and stress, and increased risk of mortality (Wilkins et al., 2010; Barsuglia et al., 2014).

There is a great need to develop non-pharmacological interventions for patients with Alzheimer’s disease as there is no current cure, and thus it is critical to help improve the quality of life of both the patient and the caregiver. Although there are several interventions that have been designed to reduce stress and emotional difficulties in the patient with Alzheimer’s disease and their caregiver, many of the interventions require substantial investment of resources from both the caregiver and patient (i.e., time, energy, and commitment). For instance, there have been successful interventions that involve psychotherapy and education, and physical activity, among other interventions (Almeida et al., 2019; Cheng et al., 2019). Because the family caregiver typically experiences physical, emotional, and mental exhaustion due to their caregiving role, they may have little time, energy, or motivation to commit to an intensive intervention.

In this review, we propose the utility of examining the effectiveness of interpersonal synchrony for supporting relational well-being and prosocial behavior in caregiver-care recipient relationships, in particular Alzheimer’s disease and related dementias. Utilizing interpersonal synchrony may be a quick and straightforward approach that requires few resources as caregivers and patients could engage in such simple behaviors as walking together, or tapping their fingers in synchrony. The purpose of this review is to highlight key findings in the literature on interpersonal synchrony, relational well-being, and prosocial behavior in healthy adults to provide a case for the importance of examining the utility in the caregiver-care recipient relationship.

This article is organized in the format of a narrative review in which we have synthesized the literature on this topic in healthy adults that examines relationships among interpersonal synchrony and prosocial behavior and well-being through the lens of psychological, neuroimaging, and neuroendocrine approaches. We then present information about the few studies that have examined this relationship in patients with Alzheimer’s disease and related dementias and/or their caregivers. Finally, we identify gaps in the literature examining interpersonal synchrony, prosocial behavior, and well-being in patients with Alzheimer’s disease and related dementias and their caregivers. This narrative review will serve as a first step in understanding the utility of interpersonal synchrony for facilitating prosocial behavior and well-being in patients with dementia and their caregivers in older adulthood.

## Interpersonal Synchrony in Healthy Adults

### Interpersonal Synchrony: Relationships With Positive Mood and Affiliation

Engaging in matching actions or behaviors can facilitate interpersonal synchrony between individuals at the level of neural activity, neuroendocrine responses, and emotions (see Table 1). In fact, the simple act of matching breathing or tapping fingers at the same pace (i.e., forms of interpersonal synchrony) has been shown to improve positive emotions, as well as affiliative feelings and prosocial behavior toward others (Hove and Risen, 2009; Ramseyer and Tschacher, 2011; Valdesolo and DeSteno, 2011; Tunçgenç and Cohen, 2016; Bhat et al., 2017; Stupacher et al., 2017; Galbusera et al., 2019; Oesch, 2019).

**Table 1:** Interpersonal synchrony in healthy adults.

Authors	Participants	Measurements	Conditions	Findings
Bamford and Davidson, 2019	237 college students filled out surveys; 21 completed the task, 18–23 years (8 women, 13 men)	Surveys (BFAS, EQ-short) and task (RT)	Correlational	↑ EQ-short scores associated with faster entrainment of movement to new rhythmic stimulus
Bhat et al., 2017	15 younger adults (8 men, 7 women), 19–27 years, *M_age_* = 22.6 years	Imaging (fNIRS), reach and cleanup task	Watch, do, together	Greater activation in together versus do conditions in R precentral and postcentral, fronto-parietal cortices, inferior parietal gyri, supramarginal and angular gyri
Birditt et al., 2017	156 middle-age adults (56% women), 45–65 years, *M_age_* = 55.9 years	Saliva sampling (cortisol): 4 days of saliva collection 3x’s a day (waking, 30 minutes after waking, and bedtime); self-report measures (daily experiences: contact, negative interactions, avoidance of negative interactions, negative thoughts, positive interactions)	Correlational	More negative interactions with parents than adult children; contact and negative interactions associated with DCS for interactions with adult children
Galbusera et al., 2019	66 adults (45 women, 21 men), *M_age_* = 27.5 years	Movement task (BCT); emotion measures: PANAS, S-DERS, SSCCS; intrapersonal and interpersonal synchrony through assessment of movement (WBB)	Within person design; paired with another participant	Greater INS ↓ self-regulation of affect and greater positive affect
Goldstein et al., 2018	22 heterosexual couples (44 participants), 23–32 years, women *M_age_* = 25.6, men *M_age_* = 26.4	Brain activity (dual-electroencephalogram-EEG) in both partners; numerical pain scale; only the women in the sample received the pain stimulation	Six counterbalanced conditions: no-pain-alone, pain-alone, partner touch–no-pain, partner no-touch–no-pain, partner touch–pain, and partner no-touch–pain	When the women received pain stimulation while holding their male partner’s hands, there was increased coupling in central regions of the womens’ brains and in the right hemisphere in the mens’ brains
Hove and Risen, 2009	165 college students from Cornell University	Tapping task, surveys	Synchronous, asynchronous, and no tap	Interpersonal synchrony predicted affiliation ratings
Ikeda et al., 2017	97 college students (32 women, 65 men), 18–26 years, *M_age_* = 21 years	Imaging (fNIRS), task: group walking, group stepping	Group walking, group stepping (both tasks with and without metronome)	↑ INS brain activity when walking with metronome beat than without beat
Liu et al., 2013	19 heterosexual couples, women *M_age_* = 39.1 years, and men *M_age_* = 41.4 years	Saliva sampling of cortisol (CAR, DCS), surveys assessing spousal support, strain, and disagreement adapted from MIDUS study	Correlational	DCS synchronized between couples, greater CAR synchrony in couples reporting high marital strain and disagreement, ↑ spousal support linked to CAR stability
Pauly et al., 2020	Study 1: 85 couples aged 60–87 years; Study 2: 77 couples aged 66–85 years)	Saliva samples 5–7x’s per day for 7 days (cortisol hormone), questionnaires	Correlational	Significant dyadic covariation in cortisol (synchrony); Cortisol synchrony ↑ when reported prior positive socioemotional partner interactions
Roberts et al., 2013	17 male police officers and their wives (34 total participants), women *M_age_* = 33.8 years, and men *M_age_* = 35.8 years	Marital adjustment test (measuring marital satisfaction), Police Stress Survey, reporting stress levels in daily diaries, behavioral coding of emotional behavior towards partner during conversation	Correlational	↑ job stress officers ↑ synchrony with wives’ affection and less synchrony with wives’ hostility; Wives showed ↑ synchrony with officers’ hostility and less synchrony with officers’ affection
Shrout et al., 2020	43 healthy adult married couples (86 individuals), adults *M_age_* = 38.2 years (*SD* = 8.2, range = 24–61)	Salivary cortisol (5 samples per participant); Perceived Stress Scale, 20-min marital problem discussion, Rapid Marital Interaction Coding System (RMICS)	Comparison of cortisol levels before and after the marital problem discussion and associations with other measures	Individuals with partners who had ↑ perceived stress, flatter cortisol slopes; Individuals with partners who had ↑ perceived stress, had ↑ cortisol levels after conflict discussion at these time-points (post 30-min, 1 hour, and 4 hours)
Stupacher et al., 2017	40 college students (20 women, 20 men), *M_age_* = 23.7 years	Explicit measure: surveys assessing likability of the experimenter; Implicit measure: measuring helping behavior (picking up pencils that were dropped)	Tapping to the beat of a metronome or music with an experimenter who tapped synchronously or asynchronously	Synchronous tapping with the experimenter to music (but not a metronome) associated with greater prosocial behavior
Valdesolo and DeSteno, 2011	69 individuals	Rhythmic tapping task, surveys measuring similarity and likability	Synchrony, asynchrony	Participants showed ↑ compassion and altruistic behaviors in synchrony condition

Studies are listed in alphabetical order. BCT, Body Conservation Task; BFAS, Big Five Aspects Scale; CAR, cortisol awakening response; DCS, diurnal cortisol slope; EQ-short, Empathy Quotient Short Form; INS, interpersonal synchrony; MEG, magnetoencephalography; MIDUS, Midlife in the United States Study; PANAS, Positive and Negative Affect Schedule; R, right; RT, rhythmic entrainment task; S-DERS, State Difficulties in Emotion Regulation Scale; SSCCS, State Self-control Capacity Scale; WBB, whole body behavioral.

There is consistent evidence in healthy younger adults that engaging in interpersonal synchrony is associated with higher levels of positive mood and affiliation (Hove and Risen, 2009; Galbusera et al., 2019). In a study by Hove and Risen (2009), the authors investigated the degree to which interpersonal synchrony was associated with self-reported feelings of affiliation (i.e., liking) in a sample of students. The task included the following conditions: (1) tapping synchronously with a metronome and experimenter, (2) tapping asynchronously with an experimenter, or (3) not tapping. In Experiment 1 (*N* = 44), the researchers found that greater interpersonal synchrony was associated with higher ratings of likeability toward the experimenter. Similarly, in Experiment 2 (*N* = 74), the researchers found that even after controlling for baseline levels of likeability toward the experimenter, greater interpersonal synchrony was associated with greater liking toward the experimenter. A study by Galbusera et al. (2019) investigated the role of interpersonal synchrony in improving positive mood in a group of 66 younger adults. In this task, participants were randomly paired with another participant (a stranger) and performed full body dyadic movements together. They found that increased levels of interpersonal synchrony were associated with higher levels of positive affect. Taken together, these studies point to an association between interpersonal synchrony and positive or affiliative mood in younger adults.

Engaging in interpersonal synchrony has also been linked to greater prosocial behaviors, or helping behaviors toward others (Valdesolo and DeSteno, 2011; Stupacher et al., 2017). A study by Stupacher et al. (2017) examined the influence of synchronous vs. asynchronous motor behavior on prosocial behavior. Specifically, 40 younger adults participated in this study which assessed the influence of synchronous and asynchronous motor tapping with an experimenter when listening to music vs. a metronome on prosocial behavior. The researchers found that when participants tapped along synchronously with the experimenter while listening to music, the participant showed greater prosocial behavior in the form of picking up more pencils that were dropped by the experimenter, in comparison to the other conditions. A study by Valdesolo and DeSteno (2011) examined the link between interpersonal synchrony and both affiliative feelings and prosocial behavior in 69 people. Participants were assigned to either a synchronous condition or an asynchronous motor tapping condition in which a confederate either tapped in sync with the participant or tapped asynchronously. After the tapping portion of the study, the participant observed the confederate experience a moral transgression. The primary results were that participants who underwent the interpersonal synchrony condition not only felt more compassion for the confederate, but also decided to help them more frequently and for a longer amount of time. In summary, these research studies add to the evidence for a positive association between interpersonal synchrony and affiliative feelings/behavior.

### Interpersonal Synchrony: Neural and Neuroendocrine Correlates

Engaging in interpersonal synchrony has also been linked to coupling, or covariation in intensity or frequency between two individuals at both the neural and neuroendocrine levels (Liu et al., 2013; Goldstein et al., 2018). In couples, the experience of affiliative touch has been associated with brain coupling between partners and a reduction in the experience of pain (Goldstein et al., 2018). Specifically, Goldstein and colleagues investigated neural synchrony between younger adult couples during the experience of pain while the partners held hands and underwent electroencephalography (EEG; Goldstein et al., 2018). The researchers found that there was, “brain-to-brain coupling,” in the alpha-mu band during the pain/hand holding condition located in central regions of the brain for the person who experienced the pain, and in the right hemisphere of the person who was the pain observer. In fact, greater brain coupling within this network was associated with the degree of pain relief experienced and the pain observer’s level of empathic accuracy (i.e., the ability to detect the thoughts, feelings, and intentions of others; Goldstein et al., 2018).

In a study by Bhat et al. (2017), the participant was asked to watch the tester cleanup the blocks and at the same time mirror their actions with each block so that they were doing the task in synchrony while undergoing functional near infrared spectroscopy (fNIRS). In two other conditions, they either only watched the tester, or did the task on their own. The authors found greater brain activity in the interpersonal synchrony condition in the right/ipsilateral fronto-parietal region and inferior parietal cortices when compared to the condition where the participant did the task on their own.

Brain systems associated with action observation and the experience of reward help to support multiple aspects of interpersonal synchrony (Stupacher et al., 2017; Oesch, 2019). Brain regions associated with ability to experience others emotions, or mimic others’ actions have also been cited as an important factor in interpersonal synchrony for promoting feelings of affiliation (Hove and Risen, 2009; Liu et al., 2013; Rabinowitch and Knafo-Noam, 2015; Bamford and Davidson, 2019). One of the functions of these regions is to support understanding of others’ intentions and goals (Praszkier, 2016). Regions important for the experience of self and thinking about others’ have shown some neuronal overlap, and thus may support processes of interpersonal synchrony (Hove and Risen, 2009).

Interpersonal synchrony can also facilitate neuroendocrine synchrony which is defined as covariation in neuroendocrine hormone levels between two or more people who are engaged in a common task, or who spend significant amounts of time together (Liu et al., 2013; Shrout et al., 2020). This can occur in the context of couples who live together and face the challenges of balancing external stressors in the context of their relationship with each other, or even groups such as athletic teams during a sporting event (Liu et al., 2013; Roberts et al., 2013). Because the focus of this review is in the examination of impacts of interpersonal synchrony in the context of caregiver-care recipient dyads, we will focus on hormones involved in the stress response, in particular cortisol (Ebner et al., 2015; Mu et al., 2016).

Liu et al. (2013) examined neuroendocrine synchrony in 19 heterosexual middle-aged couples for the stress-related hormone cortisol over 4 days. They found that the diurnal slope of cortisol (i.e., change in the cortisol hormone over the course of the day) was synchronized within the couples. Another study by Pauly et al. (2020) also examined cortisol synchrony, but specifically focused on healthy older adult couples across two studies (Study 1: *N* = 85 couples; Study 2: *N* = 77 couples). The study entailed completing saliva samples 5–7 times a day over 7 days. The authors found evidence for cortisol synchrony within the couples in the form of significant dyadic covariation in both of the studies. Of note, the authors found that the cortisol synchrony within the couple was higher in the case that the partners reported previous interactions that were positive in nature.

Another study investigated cortisol synchrony in couples in the context of an acute lab setting. Specifically, Shrout et al. (2020) investigated 43 healthy adult couples who ranged from 24 to 61 years of age. In the lab, the couples completed a marital problem solving task and had saliva samples taken before and after the marital conflict to assess cortisol levels. They also completed a standard measure of perceived stress (the Perceived Stress Scale). The authors found that individuals whose partners reported more stress had flatter cortisol slopes than individuals with less stressed partners. Furthermore, individuals with more stressed partners had higher cortisol levels after the conflict discussion than those with partners who were less stressed. Taken together, these studies demonstrate cortisol synchrony within couples in the home and lab setting, and indicate that if one partner is experiencing stress, it is likely to impact the other individual’s cortisol levels. These results have significant implications for the caregiver-care recipient context, especially in the case of caregivers who live with individuals with Alzheimer’s disease and related dementias and may face daily stressors while providing care.

## A Case For Examining the Utility of Interpersonal Synchrony in Alzheimer’s Disease and Related Dementias

Although the link between interpersonal synchrony and affiliation has been well-established in healthy adults, little is known about interpersonal synchrony in the context of the caregiver-care recipient relationship, especially as it relates to older patients with dementia. In general, interpersonal synchrony has been associated with symptom reduction in chronic illness and has been found to be a useful therapeutic tool (Güney et al., 2015; Hove and Keller, 2015). Thus, it is surprising that, given the beneficial effects of interpersonal synchrony on well-being and prosocial behaviors in healthy populations, very little research has been done in the context of caregiver-care recipient interactions in older adulthood.

Addressing this gap in the literature is critical because Alzheimer’s disease and related dementias are a significant public health concern since there is no cure, and there is a great need for developing interventions to target modifiable factors to improve patients’ quality of life. A key factor that can put great strain on the caregiver-care recipient relationship in Alzheimer’s disease and related dementia is the presence of neuropsychiatric symptoms (NPS). Of note, a large proportion of patients with dementia (up to 88% of cases) experience NPS, such as apathy and depression, which are associated with poorer patient cognition, functional outcomes, greater morbidity and mortality, and higher cost of care than patients without NPS (Mega et al., 1996; Lyketsos et al., 2000; Steffens et al., 2005).

However, researchers have identified a potentially modifiable factor that is associated with lower levels of NPS symptoms in AD. Specifically, caregiver-care recipient dyads that have higher levels of closeness report lower NPS symptoms (Vernon et al., 2019). Furthermore, closer caregiver-care recipient relationships have also been associated with higher levels of both quality of life and well-being (Burgener and Twigg, 2002). In healthy adults, engaging in interpersonal synchrony can increase positive feelings such as liking and affiliation toward the other person which in turn could increase levels of closeness. Therefore, it may be useful to examine the utility of interpersonal synchrony to help increase closeness levels in the caregiver-care recipient relationship in Alzheimer’s disease and related dementias.

Another important factor that has been associated with greater caregiver burden is decreased functioning by the patient with AD in the area of social and emotional processing (Martinez et al., 2018). In particular, patients with AD typically have a reduced capacity to recognize others’ emotions (Güntekin et al., 2019). Furthermore, evidence for this was found in a meta-analysis of studies of patients with AD that found strong evidence for a decrease in patients’ abilities to identify the thoughts, feelings, and motivations of others, a process called cognitive empathy or theory of mind (Demichelis et al., 2020). On the other hand, patients with AD show relative preservation in their ability to experience emotions such as compassion and sympathy towards others, a process called emotional empathy (Demichelis et al., 2020). The patients’ decreased capacity to understand others’ motivations and feelings could lead to misunderstandings with the caregiver and cause unnecessary stress for the patient and increased caregiver burden.

Patients with behavioral variant frontotemporal dementia (bvFTD) also suffer from declines in social and emotional processing, and thus the utility of interpersonal synchrony in this population should be examined. A study by Barsuglia et al. (2014) found that patients with bvFTD showed significant deficits in social synchrony and interpersonal bonding (Barsuglia et al., 2014). These differences in social synchrony and interpersonal bonding were associated with poor awareness and adherence to social boundaries, as well as diminished interest in activities that were once meaningful to the individual (Barsuglia et al., 2014). This study points to the negative impact of reduced socioemotional processing on the patient with bvFTD, and may also increase caregiver burden. Interpersonal synchrony may be useful in boosting affiliative mood and behavior between the caregiver and patient.

In summary, patients with Alzheimer’s disease and related dementias experience a decreased capacity to detect others’ thoughts and emotions, as well as other difficulties with social processing which could lead to misunderstandings and the development of distrust in the family caregiver. Consequently, it would be relevant to consider the utility of using interpersonal synchrony to help facilitate trust and relationship satisfaction between the patient and the caregiver which ultimately may foster overall well-being in the caregiver-care recipient dyad.

## The Limitations of Interpersonal Synchrony

Though the use of interpersonal synchrony has been shown to facilitate affiliation in the context of relationships, there are important limitations to consider. In particular, careful investigation is needed to determine in which situations interpersonal synchrony is most useful, and the implications of the overuse of synchrony (Liu et al., 2013; Roberts et al., 2013; Wallot et al., 2016; Galbusera et al., 2019). For instance, too much interpersonal synchrony can cause a tempering of self-regulation abilities in healthy adults (Galbusera et al., 2019). However, the exact cutoff for how much synchrony is too much remains unclear (Galbusera et al., 2019). Additionally, there are certain social contexts where the use of interpersonal synchrony can be disadvantageous (Roberts et al., 2013; Wallot et al., 2016). For instance, in couples where one partner works in a high stress environment, greater interpersonal synchrony is associated with marital disharmony (Roberts et al., 2013). However, compensatory emotional states were found to significantly improve emotional state and productivity in partners, with some researchers arguing that compensatory states should be considered as another form of synchrony (Roberts et al., 2013; Wallot et al., 2016).

Interpersonal synchrony can be influenced by one’s living environment (i.e., living with a spouse for decades), and can be facilitated through interventions focused on motor synchrony (i.e., tapping, or moving to music) or affiliative touch. Further research is needed to explore the impacts of naturally occurring interpersonal synchrony in the home environment that affects neural and hormonal systems related to social bonding and emotion. For instance, the effects of living with a spouse on interpersonal synchrony could be extremely variable and be influenced by such important factors as the relationship duration and closeness, and individual factors, such as mental and physical health, stress, emotional reactivity, and regulation. Interventions designed to boost synchrony (e.g., motor synchrony) could modulate levels of interpersonal synchrony in caregiver-care recipient dyads. Yet, much research is needed to understand the specificity, sensitivity, and timeline of different types of interpersonal synchrony interventions on relational well-being. In sum, more research is needed to investigate which applications of interpersonal synchrony are most useful in the context of caregiver-care recipient relationships, and the degree to which these interventions may interface with other interventions designed to support caregiver-care recipient wellness, such as psychotherapy, exercise interventions, and meditation.

## Conclusion and Future Directions

In conclusion, interpersonal synchrony can elicit pro-social behavior and affiliative feelings in healthy adults. Interpersonal synchrony through synchronized motor movements may be a straightforward process that could be utilized with couples and groups. Thus, it is crucial to better understand what benefits could be derived from the development of interpersonal synchrony between caregivers and care recipients, in particular, patients with Alzheimer’s disease and related dementias. The use of interpersonal synchrony may help to foster positive feelings between the patient and caregiver which in turn can support higher quality and rewarding relationships. However, it is critical to understand both the advantages and disadvantages of the use of interpersonal synchrony on caregiver-care recipient well-being. Future studies may investigate which types of interpersonal synchrony are most effective in boosting relationship satisfaction, prosocial behaviors, and well-being in caregiver-care recipient relationships in older adulthood.

## Author Contributions

AG, JB, and VM contributed to the literature review, writing and editing of the manuscript. All authors contributed to the article and approved the submitted version.

### Conflict of Interest

During manuscript development, JB served as a consultant for Boys Town National Research Hospital in the form of a faculty mentor to a Research Scientist on staff for the time period (11/1/2019 – 10/31/2020). JB is a Section Editor at Current Behavioral Neuroscience Reports and is part of the Editorial Board at the Journal of Gerontology Psychological Sciences. As part of her service outreach, JB is also part of the Nebraska Caregiver Coalition, the Lifespan Respite Advisory Group for the state of Nebraska, and the Aging with Passion and Purpose Conference Planning Committee.

The remaining authors declare that the research was conducted in the absence of any commercial or financial relationships that could be construed as a potential conflict of interest.
